# Celastrol attenuates arterial and valvular calcification via inhibiting BMP2/Smad1/5 signalling

**DOI:** 10.1111/jcmm.15779

**Published:** 2020-09-20

**Authors:** Zhongping Su, Pengyu Zong, Ji Chen, Shuo Yang, Yihui Shen, Yan Lu, Chuanxi Yang, Xiangqing Kong, Yanhui Sheng, Wei Sun

**Affiliations:** ^1^ Department of Cardiology The First Affiliated Hospital of Nanjing Medical University Nanjing China; ^2^ Department of translational medicine Collaborative Innovation Center for Cardiovascular Disease Translational Medicine Nanjing Medical University Nanjing China

**Keywords:** BMP2/Smad1/5 signalling, celastrol, high calcium, vascular and valvular calcification

## Abstract

Vascular calcification is an important risk factor for the mortality and morbidity in chronic kidney disease (CKD). Unfortunately, until now there is no certain medication targeting vascular calcification in CKD. In this study, we explored the inhibitory effect of celastrol on high calcium–induced vascular calcification and the underlying molecular mechanisms. Cell proliferation assay showed that celastrol inhibited aortic valve interstitial cell (VIC) and vascular smooth muscle cell (VSMC) proliferation when its concentration was higher than 0.6 μmol/L. 0.8 μmol/L celastrol inhibited the expression of osteogenic genes and calcium deposition induced by high‐calcium medium in both AVICs and VSMCs. In mouse vascular calcification model induced by adenine combined with vitamin D, alizarin red and immunostaining showed that celastrol inhibited pro‐calcification gene expression and calcium deposition in aortic wall and aortic valve tissues. At the molecular level, celastrol inhibited the increase of BMP2, phosphorylated Smad1/5 (p‐Smad1/5) and non‐phosphorylated β‐catenin (n‐p‐β‐catenin) induced by high‐calcium medium both in vitro and in vivo. Also, BMP2 overexpression reversed the anti‐calcification effects of celastrol by recovering the decrease of p‐Smad1/5 and n‐p‐β‐catenin. Furthermore, celastrol prevented the up‐regulation of BMPRII and down‐regulation of Smad6 induced by high calcium, and this protectory effect can be abolished by BMP2 overexpression. In conclusion, our data for the first time demonstrate that celastrol attenuates high calcium–induced arterial and valvular calcification by inhibiting BMP2/Smad1/5 signalling, which may provide a novel therapeutic strategy for arterial and valvular calcification in patients with CKD.

## 1 INTRODUCTION

Cardiovascular diseases is one of the main complications in patients with chronic kidney disease (CKD), accounting for over 50% of all deaths in patients with end‐stage renal disease (ESRD).^1^ Vascular calcification, including aortic valve and artery calcification, is an important risk factor for cardiovascular mortality in ESRD.^2^, ^3^ Along with the compromised renal function, many factors such as dysregulated calcium‐phosphate metabolism and increased systematic inflammation promoted the progression of cardiovascular calcification.^4^ Vascular calcification usually accelerates in CKD patients and become even worse in end‐stage patients relying on maintenance haemodialysis.^5^ Many studies have identified that the progression of artery and aortic valve calcification is an active and highly adjustable biological process, which is associated with inflammation, oxidative stress, extracellular matrix remodelling, calcium‐phosphorus metabolism abnormalities, lipid deposition and ectopic mineralization.^6^, ^7^, ^8^ Vascular smooth muscle cells (VSMCs) play a critical role in mediating the progression of vascular calcification by undergoing osteogenic‐like differentiation and secreting matrix vesicles, which usually serve as the nuclei for calcium‐phosphate deposition.^9^ Aortic valve interstitial cells (AVICs) promote valvular calcification in a similar way.^10^

Molecules in transforming growth factor β (TGF‐β) and Wnt signalling family are critical in the development of bone and cartilage. They also play essential roles in osteogenic differentiation of AVICs and VSMCs during vascular calcification. Bone morphogenetic protein 2 (BMP2), a member of TGF‐β superfamily, has been recognized as one of the most important mediator of cardiovascular calcification.^11^ BMP2 has been identified as a key molecule involved in ectopic bone formation and stimulated mineralization in soft tissue, and is also important in determining the phenotype profile of osteogenic transdifferentiation of VSMCs and AVICs.^12^ BMP2 signalling is initiated by binding to its specific receptors, which leads to in the phosphorylation and nuclear translocation of Smad1/5.^13^ Translocated Smad1/5 then modulates the expression of downstream osteogenic genes. Therefore, inhibiting the pathological increase of BMP2 may be a potential strategy in the pharmacological intervention of vascular calcification in CKD.

Natural herbal chemicals are important sources for developing novel medications. An ancient herbal medicine, thunder god vine (TGV) has been used in the treatment of chronic inflammatory diseases in China since two thousand years ago.^14^ Celastrol (CEL, C_29_H_38_O_4_), a pentacyclic triterpene derived from the root extracts of TGV, is a potent immunosuppressive, anti‐inflammatory and anticancer agent.^15^, ^16^ Several studies showed that CEL had a significant protecting effect on many diseases, for example glomerular diseases,^17^ Alzheimer's disease,^18^ systemic lupus erythematosus,^19^ rheumatoid arthritis,^20^ gliomas^21^ and prostate cancer.^22^ In addition, the latest study on CEL has demonstrated that it is able to ameliorate some metabolic diseases, such as obesity, or suppress cardiac and renal fibrosis.^23^, ^24^, ^25^ However, the functional role of CEL in cardiovascular calcification and relevant osteogenic changes has not yet been clarified.

In our study, we identified the potential molecular mechanisms of the anti‐calcification effect of CEL in vascular calcification. We show for the first time that CEL alleviates aortic valve and aortic artery calcification induced by high calcium by suppressing the osteogenic transdifferentiation of AVICs and VSMCs. CEL significantly inhibits the expression of BMP2 and BMPRII, and preserves the expression of Smad6, which further results in the inhibition of Smad1/5 and activation of Wnt/β‐catenin.

## 2 MATERIALS AND METHODS

### 2.1 Cell culture

Porcine aortic valve interstitial cells (pAVICs) were isolated as described previously.^26^ The cells were cultured in Dulbecco's modified Eagle's medium (DMEM, Gibco) supplemented with 10% foetal bovine serum (FBS, Gibco), penicillin (100 U/mL) and streptomycin (100 µg/mL). Human aortic vascular smooth muscle cells (HAVSMCs) were purchased from the ATCC (no. CRL‐1999) and were incubated in DMEM containing 10% FBS and 1% streptomycin and penicillin. Calcification was induced by conditioned medium (CM, DMEM containing 5% FBS, 200 mmol/L calcium and 200 mmol/L phosphate) for 48‐72 hours. CEL (C0869, Sigma, Germany) was dissolved in DMSO. Cells were treated with 0.8 μmol/L CEL (final concentration of DMSO less than 1‰) with or without CM for 48‐72 hours and then collected for further measurements.

Cells were cultured to 60%‐70% confluency and then infected with recombinant adenovirus expressing human BMP2 (GeneChem) according to the manufacturer's instructions. Three concentrations of adenovirus BMP2 vector, 1 × 10^7^ pfu/mL, 2 × 10^7^ pfu/mL and 3 × 10^7^ pfu/mL, were used to induce BMP2 overexpression.

### 2.2 Real‐time cell analysis (RTCA)

Real‐time monitoring of the proliferation of pAVICs and HAVSMCs were performed by using RTCA (a label‐free dynamic technology). An E‐Plate assay plate was used to seed the cell suspensions (2000‐3000 cells/well, 100 μL) and was placed in the incubator to monitor by RTCA. After 24 hours, serum‐starved cells were incubated with various concentrations of CEL for 48 hours and the cytotoxicity of CEL was assessed using RTCA Analyzer (ACEA Biosciences).

### 2.3 Measurement of plasma concentration of CEL

The whole blood was collected at 0.25, 0.5, 1, 2, 3, 4, 6, 10, 12, 15, 18 and 24 hours after subcutaneous injection of CEL (2 mg/kg) in mice. Then, centrifugation at 3000 r/min for 10 minutes was used to isolate plasma for further study. The plasma was dried by nitrogen and dissolved in methanol (CNW Technologies GmbH). U‐HPLC (Thermal Fisher) was coupled to a Q‐Exactive Orbitrap™ spectrometer (Thermal Fisher) with an electrospray ionization (ESI) source for detection. The solvents consisted of deionized H_2_O (Aladdin) with 0.1% formic acid and methanol with 0.1% formic acid. The gradient started from 30% methanol and increased rapidly to 95% at 4 minutes, and decreased to 30% at 5 minutes till the end. Further analysis was carried out using HPLC‐MS/MS for more sensitive and specific measurements.

### 2.4 Animal experiments

A total of 35 specific pathogen‐free (SPF) male C57/BL6 mice aged 6 weeks were provided by the Model Animal Research Center. CKD model was induced by adenine diet. These mice were randomly divided into two groups (6‐10 mice per group) and fed either normal chow or adenine for 3 weeks. To induce calcification, mice were intraperitoneally injected with olive oil or vitamin D (Vit D, 8.75 mg/kg/day, Sigma‐Aldrich) for 10 days. The vitamin D we used is calcitriol. In addition, mice were subcutaneously injected with CEL (2 mg/kg/day) or vectors for 14 days starting from Vit D injection. Under anaesthesia, whole blood and tissues were collected and stored under specific conditions for further experiments. All animal experiments were approved by the Animal Use and Care Committee.

### 2.5 Calcium quantitation

Calcium deposition was measured after washing the cells with PBS. Then, 0.6 mol/L HCL was used for the de‐calcification, and calcium accumulation was determined in the supernatants using QuantiChrom™ Calcium Assay Kit (Bioassay Systems; No. DICA‐500). The protein concentration of remaining cells was determined by a BCA protein assay kit (Thermo Fisher Scientific; No. 23225). Quantification of calcium accumulation was normalized to protein concentration of each culture. To determine calcium accumulation of aortas in vivo, segment aortic tissues were decalcified with 0.6 mol/L HCL overnight, and calcium accumulation was calculated based on the above method and normalized to micrograms Ca/mg weight.

### 2.6 Alkaline phosphatase activity assay (ALP)

Cells in each group were lysed using 0.05% Triton X‐100, frozen and thawed 3 times, and then collected. The supernatants (20 μL) were mixed with ALP reagent (100 μL) and incubated at 37°C for 15 minutes. LabAssay™ ALP Kit (Wako Chemicals; Cat No. 291‐58601) was used to measure the ALP activity in cells, according to the manufacturer's instruction. ALP activity was normalized to total protein concentration.

### 2.7 Histological analyses

Calcium deposition was assessed by alizarin red staining (Cat No. BM1853).

The cells or tissues were fixed with 4% paraformaldehyde for 10‐15 minutes, dehydrated in 95% ethanol for 30 minutes and exposed to alizarin red (pH 4.2) solution for 3‐5 minutes. The samples were washed with distilled H_2_O to remove remaining dye and then photographed.

For von Kossa staining, tissues were fixed with 4% paraformaldehyde for 10‐15 minutes and washed 2 times with PBS. Tissue frozen sections were treated with silver nitrate solution (BoMei Biotechnology) and then exposed to UV for over 20 minutes. Next, the samples were stained with eosin.

### 2.8 Immunofluorescence

Mouse tissue frozen sections were fixed with 4% paraformaldehyde (10 minutes) and permeabilized with 0.5% Triton X‐100 (10‐15 minutes). To reduce non‐specific background staining, sections were blocked with 5% bovine serum albumin for 1 hour at room temperature. Then, sections were incubated overnight at 4°C with the following primary antibodies: Runx2 (1:100; Proteintech, Cat No. 20700‐1‐AP), P‐Smad1/5 (1:100; Cell Signaling, Cat No. 13820), OPN (1:100; Bioworld Technology, Cat No. BS1264), vimentin (1:100; Abcam, Cat No. 92547) and α‐SMA (1:600; Sigma, c6198). Next, Alexa Fluor 488 goat anti‐rabbit or CyTM3 (Jackson ImmunoResearch) was incubated for 1 hour at room temperature. DAPI staining was used to observe the nucleus. Then, fluorescent images were obtained using confocal laser scanning microscope (Carl Zeiss).

### 2.9 Quantitative real‐time PCR (RT‐PCR)

Total RNA was purified from cultured cells with the TRIzol (Thermo Fisher Scientific). Reverse transcription was performed using PrimeScript Master Mix (Takara Bio). RT‐PCR was performed with TaqMan Kit (Roche) and SYBR‐Green Kit (Bio‐Rad). Levels of mRNA were quantitated using an ABI Prism 7900 system. TaqMan primers and probes were purchased from Roche: Runx2 (F, 5′‐CTTTTGGGATCCGAGCAC‐3′; R, 5′‐GGCTCACGTCGCTCATCT‐3′); OPN (F, 5′‐AATCTAAGAAGTTCCGCAGATCC‐3′; R, 5′‐CCACATGTGACGTGAGGTCT‐3′). Specific primers were as follows: Runx2 (F, 5′‐TCTCAGATCGTTGAACCTTGCTA‐3′; R, 5′‐TGGTTACTGTCATGGCGGGTA‐3′); OPN (F, 5′‐GACACGAAGGTAAAGGTGAC‐3′; R, 5′‐CTGGTGCTCGTCCTCTACTAC‐3′). All samples were analysed in triplicate, and the relative levels were normalized to β‐tubulin expression.

### 2.10 Western blot analysis

Total protein was extracted with a whole cell lysis, and its concentration was measured using a BCA protein assay kit (Thermo Fisher Scientific; No. 23225). Proteins were separated on 10%‐12% SDS‐polyacrylamide gel electrophoresis and transferred to a PVDF membrane (Millipore). The membranes were blocked with 5% bovine serum albumin and then incubated with the following primary antibodies: anti‐Runx2 (Cat No. 125561; Cell Signaling, 1:1000), anti‐GAPDH (Cat No. 5174; Cell Signaling, 1:1000), anti‐P‐Smad1/5 (Cat No. 9516; Cell Signaling, 1:1000), anti‐Smad1 (Cat No. 6944; Cell Signaling, 1:1000), OPN (Cat No. BS1264; Bioworld Technology, 1:1000), anti‐non‐p‐β‐catenin (Cat No. 19807; Cell Signaling, 1:1000), anti‐β‐catenin (Cat No. 8480; Cell Signaling, 1:1000), anti‐BMP2 (ab14933; Abcam, 1:600), anti‐Smad6 (ab13727; Abcam, 1:500), anti‐BMPR‐IA (sc5676; Santa, 1:200), anti‐BMPR‐IB (sc5679; Santa, 1:200) and anti‐BMPR‐II (sc393304; Santa, 1:200). The membranes were incubated with secondary antibodies, after washing 3 times for 10 minutes with TBST. Next, enhanced chemiluminescence reagent (Thermo Fisher Scientific) was used to detect the blots.

### 2.11 Statistical analysis

All data were presented as the mean ± SEM from three independent experiments. Statistical significance was performed using either one‐way ANOVA with a Bonferroni post hoc test or Student's *t* test. All analyses were performed with GraphPad version 5.0 software. *P* < .05 was considered statistically significant.

## 3 RESULTS

### 3.1 CEL attenuates calcification induced by calcific medium in pAVICs and HAVSMCs

**FIGURE 1:**
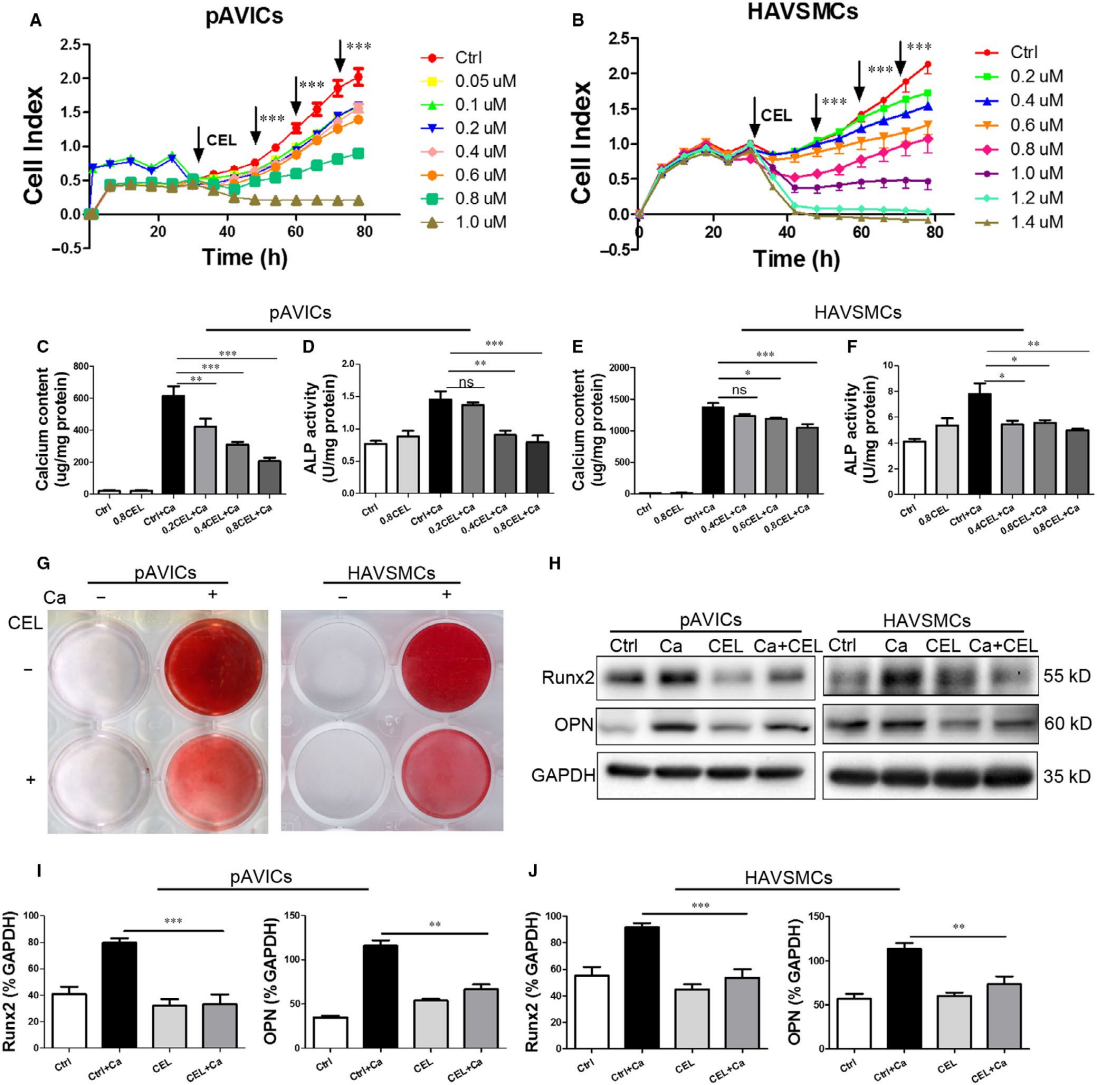
CEL attenuates calcific medium–induced calcification in pAVIC and HAVSMC calcification. A and B,Real‐time cell proliferation with CEL treatment at different concentrations by RTCA. C and E, Calcium content measured using the QuantiChrom™ Calcium Assay Kit. D and F, Alkaline phosphatase (ALP) activity assessed using the LabAssay™ ALP Kit. G, Alizarin red staining of cellular calcium deposition. H, Western blotting of Runx2 and OPN expression. I and J, Quantitative analysis of Runx‐2 and OPN expression. GAPDH served as the standard. All data are represented as mean ± SEM of at least three independent experiments. ns indicates no significant difference between the indicated columns; *P < .05, **P < .01 and ***P < .001 indicate significant differences between the indicated columns

To determine the optimal CEL (Figure [Link], [Link]A) concentration for further study, the cytotoxicity of CEL was firstly assessed using the RTCA Analyzer. Compared to the control group, no significant cell death or injury was observed after incubation for 72 hours with CEL ranging from 0.1 to 0.8 μmol/L in pAVICs and 0.2 to 1.0 μmol/L in HAVSMCs, but for both pAVICs and HAVSMCs, the cell viability was <50% at concentrations higher than 0.8 μmol/L (Figure 1A,B). There were significant differences of cell viability in different treating duration compared with the control group (Figure [Link], [Link]B,C). Calcium content and alkaline phosphatase (ALP) activity were significantly increased in pAVICs and HAVSMCs cultured with calcific medium (CM) containing high concentration of calcium and phosphorus for 3 days. CEL at the dose of 0.2‐0.8 μmol/L in pAVICs (Figure 1C,D) and 0.4‐0.8 μmol/L in HAVSMCs (Figure 1E,F) significantly inhibited the increase of calcium content and ALP activity in a dose‐dependent manner. Therefore, we chose 0.8 μmol/L for subsequent cell experiments.

To further examine the suppressive effect of CEL on calcification, we performed alizarin red staining to visualize calcium deposition in cells. Calcium deposition in pAVICs and HAVSMCs was significantly increased by the treatment with CM, and this increase was significantly inhibited by CEL (Figure 1G). To assess the effect of CEL on bone formation‐associated proteins, we examined the expression level of some osteogenic markers. We found that CEL decreased the expression levels of Runx2 and OPN, at both protein (Figure 1H‐J) and mRNA levels (Figure [Link], [Link]A,B). Taken together, our data indicate that CEL attenuates CM‐induced pAVIC and HAVSMC calcification.

### 3.2 CEL inhibits aortic valve and aortic artery calcification induced by vitamin D in CKD mouse model

To further explore the therapeutic potential of CEL for cardiovascular calcification in CKD, we build up a calcification model induced by Vit D in renal dysfunction mice induced by adenine diet. After feeding using high adenine diet for 3 weeks, the level of serum urea nitrogen (BUN) and creatine (SCr) greatly was significantly increased (Figure [Link], [Link]A,B) and accompanies with a moderate increase of serum phosphorus (Figure [Link], [Link]C). To determine dynamic changes in plasma concentration of CEL in mice, we measured the concentrations of CEL at different time‐points in plasma by liquid chromatography‐mass spectrometry technology. The results showed that the plasma concentration of CEL was higher than 8 μmol/L at 1 hour after the administration, still higher than 0.6 μmol/L at 4 hours and continuously higher than 0.4 μmol/L for next 8 hours, and the complete elimination needs about 24 hours (Figure [Link], [Link]A). Bodyweight and hepatic function monitoring were used to evaluate the toxicity of CEL to mice.^22^ CEL treatment did not lead to a significant loss of bodyweight and a significant increase in alanine transaminase (ALT)/aspartate transaminase (AST) compared with the untreated group, which indicated the dosage we chose is safe (Figure [Link], [Link]A‐C).Also, CEL treatment did not significantly altered levels of BUN or serum creatinine compared with the untreated group (Figure [Link], [Link]D,E). However, CEL seems to have a protecting effect on renal dysfunction from mice treated with Vit D (Figure [Link], [Link]D,E). Meanwhile, for mice treated with Vit D to induce vascular calcification, serum phosphorus level in the CEL group was significantly lower than those of the control group (Figure [Link], [Link]F). Our data indicated that CEL has a protective effect on renal function in a high calcium status.

**FIGURE 2:**
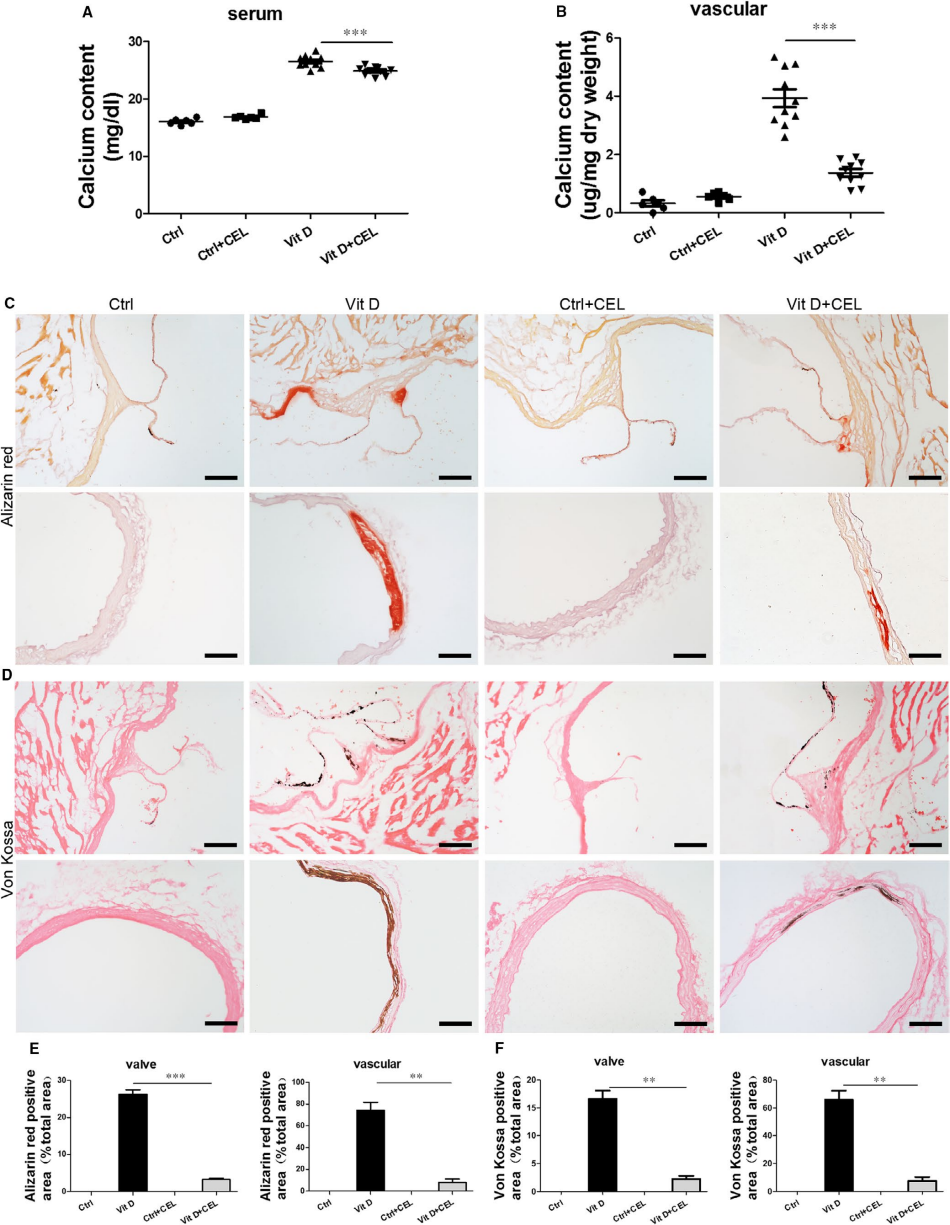
CEL inhibits aortic valve and aortic artery calcification induced by vitamin D in mice fed with adenine diet. A, Serum calcium content and B, aortic artery calcium deposition measured using the QuantiChrom™ Calcium Assay Kit. C, Calcium deposition assessed by alizarin staining in mouse aortic valve and aortic artery (scale bar = 100 µm, n = 6‐10 for each group). D, Calcium deposition assessed by von Kossa staining of mouse aortic valve and aortic artery (scale bar = 100 µm, n = 6‐10 for each group). E and F, Quantitative analysis of calcium deposition. All data are represented as mean ± SEM of at least three independent experiments. **P < .01 and ***P < .001 indicate significant differences between the indicated columns

**FIGURE 3:**
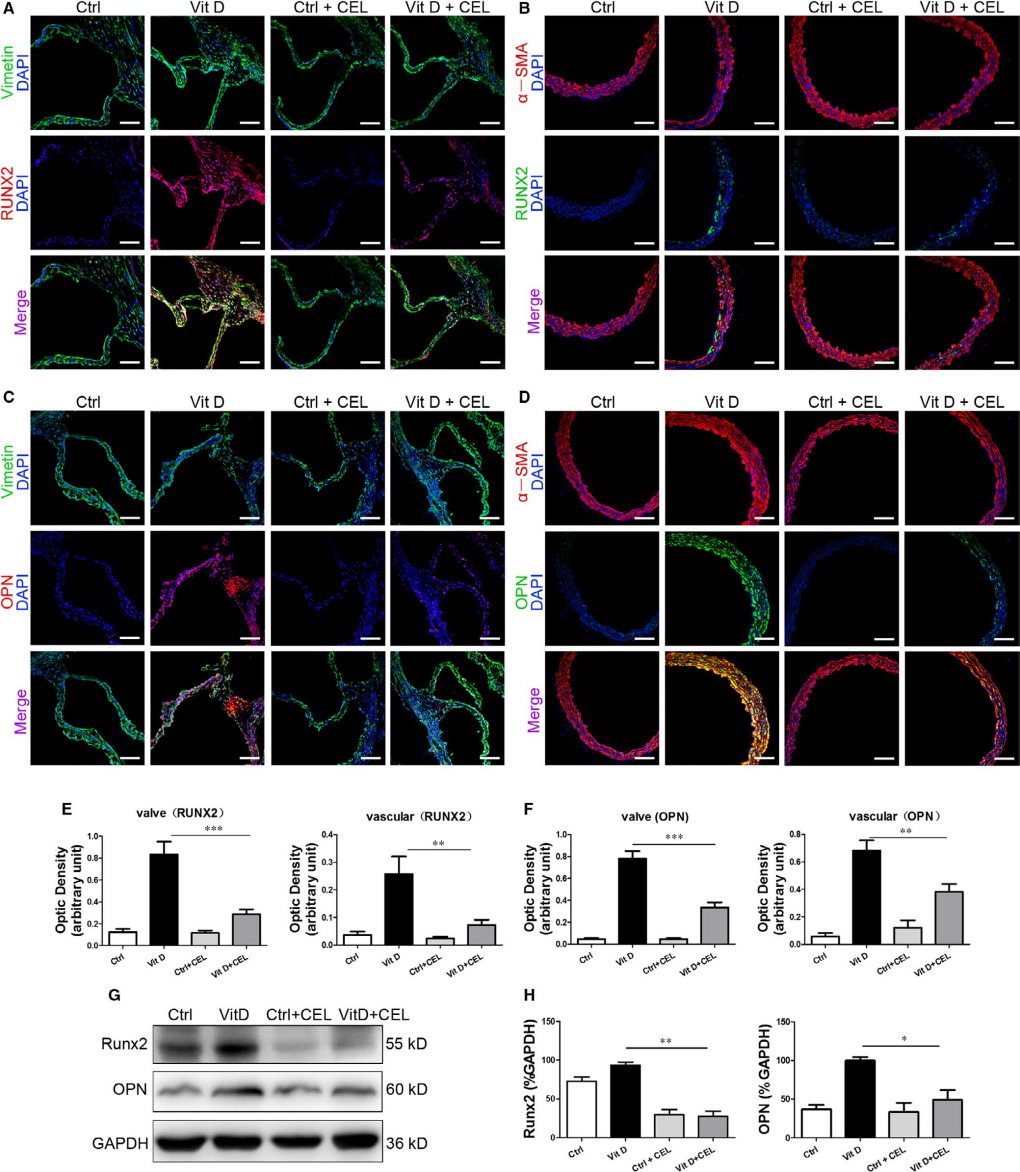
CEL inhibits the expression of Runx2 and OPN. A and B, Representative images of immunofluorescence staining of Runx2 in mouse aortic valve and aortic artery (scale bar = 20 µm, n = 6~10 for each group). C and D, Representative images of immunofluorescence staining of OPN in mouse aortic valve and aortic artery (scale bar = 20 µm, n = 6~10 for each group). E and F, Quantitative analysis of immunofluorescence intensity. G and H, Western blotting of Runx2 and OPN expression in mouse aortic artery. GAPDH served as the standard. All data are represented as mean ± SEM of at least three independent experiments. *P < .05, **P < .01 and ***P < .001 indicate significant differences between the indicated columns

We further examined the anti‐calcification effect of CEL in vivo. CEL treatment significantly decreased the calcium content in both serum and aortic artery (Figure 2A,B). Alizarin red staining and von Kossa staining showed significant calcium deposition in the aortic valve and aortic artery from mice treated with Vit D compared with control mice, and CEL treatment had a strong inhibitory effect on calcium deposition (Figure 2C‐F). We then examined the protein expression of osteogenic markers including Runx2 and OPN in vivo. Consistent with the results of cell experiments, immunofluorescence staining showed that the expression of Runx2 and OPN was significantly increased after Vit D treatment in aortic valves (Figure 3A,C,E) and aortic artery (Figure 3B,D,F), and CEL treatment inhibited this increase, which was further confirmed in aortic artery tissues using Western blotting (Figure 3G,H) and qRT‐PCR (Figure [Link], [Link]G,H). These results indicated that CEL inhibits vascular calcification both in vitro and in vivo.

### 3.3 CEL inhibits BMP2‐Smad1/5 and Wnt/β‐catenin signalling

**FIGURE 4:**
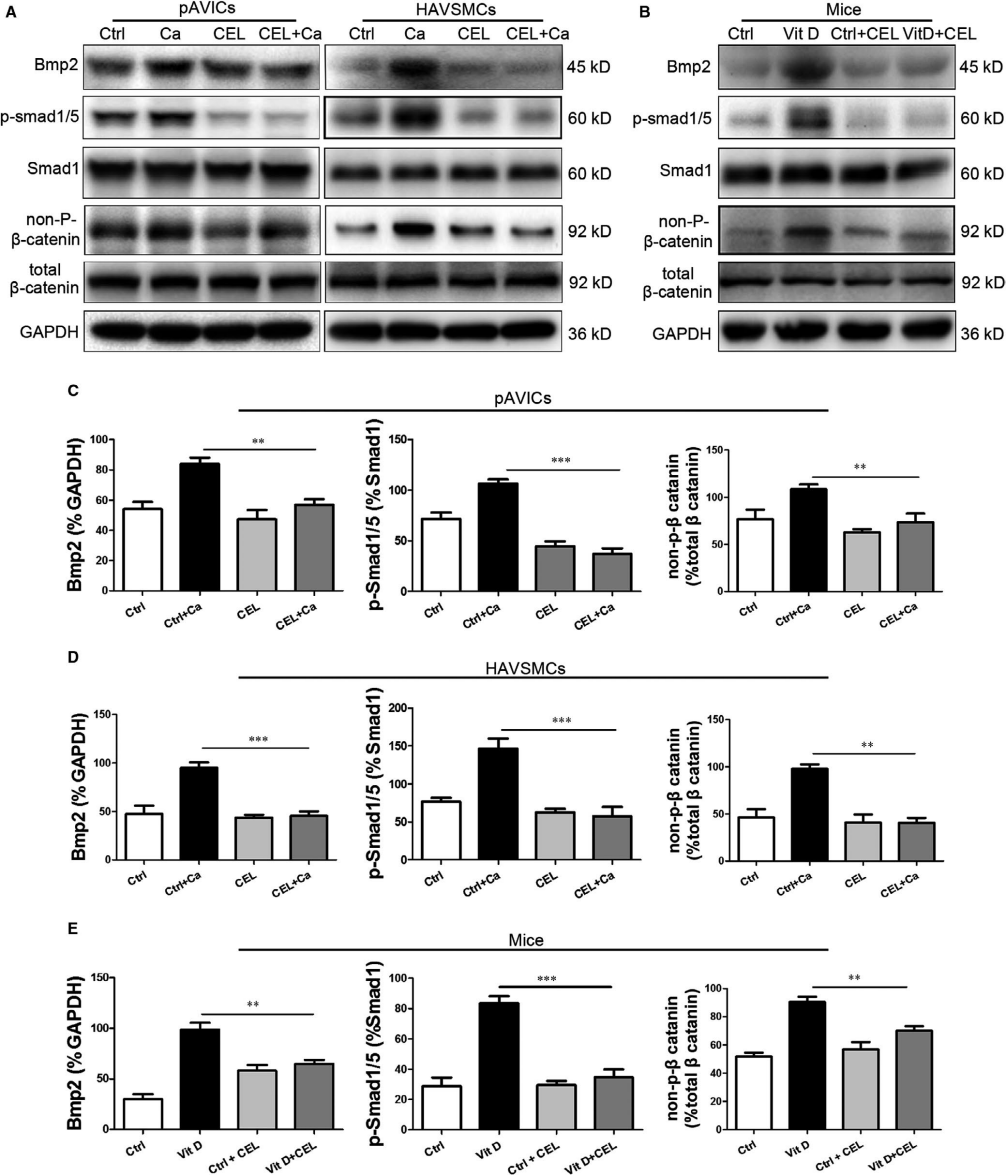
CEL down‐regulating BMP2‐Smad1/5 and Wnt/β‐catenin signalling. A, Western blotting of BMP2, p‐Smad1/5, Smad1, non‐p‐β‐catenin and total β‐catenin expression in pAVICs and HAVSMCs. B, Western blotting of BMP2, p‐Smad1/5, Smad1, non‐p‐β‐catenin and total β‐catenin expression in mice. C‐E, Quantitative analysis of protein expression. GAPDH served as the standard. All data are represented as mean ± SEM of at least three independent experiments. **P < .01 and ***P < .001 indicate significant differences between the indicated columns

As a well‐known NF‐κB signalling inhibitor, CEL exhibits a potent anti‐inflammatory effect.^16^ To explore the molecular mechanisms of CEL in inhibiting calcification, we examined the expression of p‐IκB and p‐p65 by Western blotting in pAVICs and found that CEL treatment had no effect on their expression compared with the control group (Figure [Link], [Link]A,B). Our result suggests that NF‐κB signalling may not account for the protecting effect of CEL in CM‐induced calcification. CEL improves asthma in mice by inhibiting the MAPK pathway.^27^ We also examined whether CEL has some effect on activation of p38 MAPK in pAVICs treated with CM, and Western blotting revealed that CEL markedly attenuated CM‐induced p38 phosphorylation (Figure [Link], [Link]C,D). BMP2/Smad1/5 and Wnt/β‐catenin pathways were found to be involved in the osteogenic differentiation and calcification.^12^, ^28^ The increased expression of BMP2 and non‐p‐β‐catenin in pAVICs and HAVSMCs induced by CM prompted us to investigate whether CEL suppressed calcification by targeting this two molecules, and we found that CEL effectively inhibited the increased expression of BMP2 and non‐p‐β‐catenin induced by CM (Figure 4A,C,D). Moreover, CEL had no significant effect on the expression of total β‐catenin (Figure 4A,C,D). To make clear whether CEL affects the downstream of BMP2 signalling pathway, the phosphorylation level of Smad1/5 (p‐Smad1/5) was evaluated. We found that the expression of p‐Smad1/5 expression was significantly increased after CM treatment, and this increase was suppressed by CEL (Figure 4A,C,D). However, CEL had no effect on the expression of Smad1 (Figure 4A,C,D). To further confirm these in vitro results, we examined the expression of these proteins in aortic artery tissues from mice treated with Vit D. Similarly, the expression of BMP2, non‐p‐β‐catenin and p‐Smad1/5 was significantly increased in the Vit D group, and CEL treatment effectively inhibited this increase of them in aortic arteries (Figure 4B,E). Additionally, immunostaining confirmed the inhibition of p‐Smad1/5 by CEL in both aortic valve (Figure [Link], [Link]A,B) and aortic artery (Figure [Link], [Link]C,D).

### 3.4 Overexpression of BMP2 reverses the anti‐calcification effect of CEL

**FIGURE 5:**
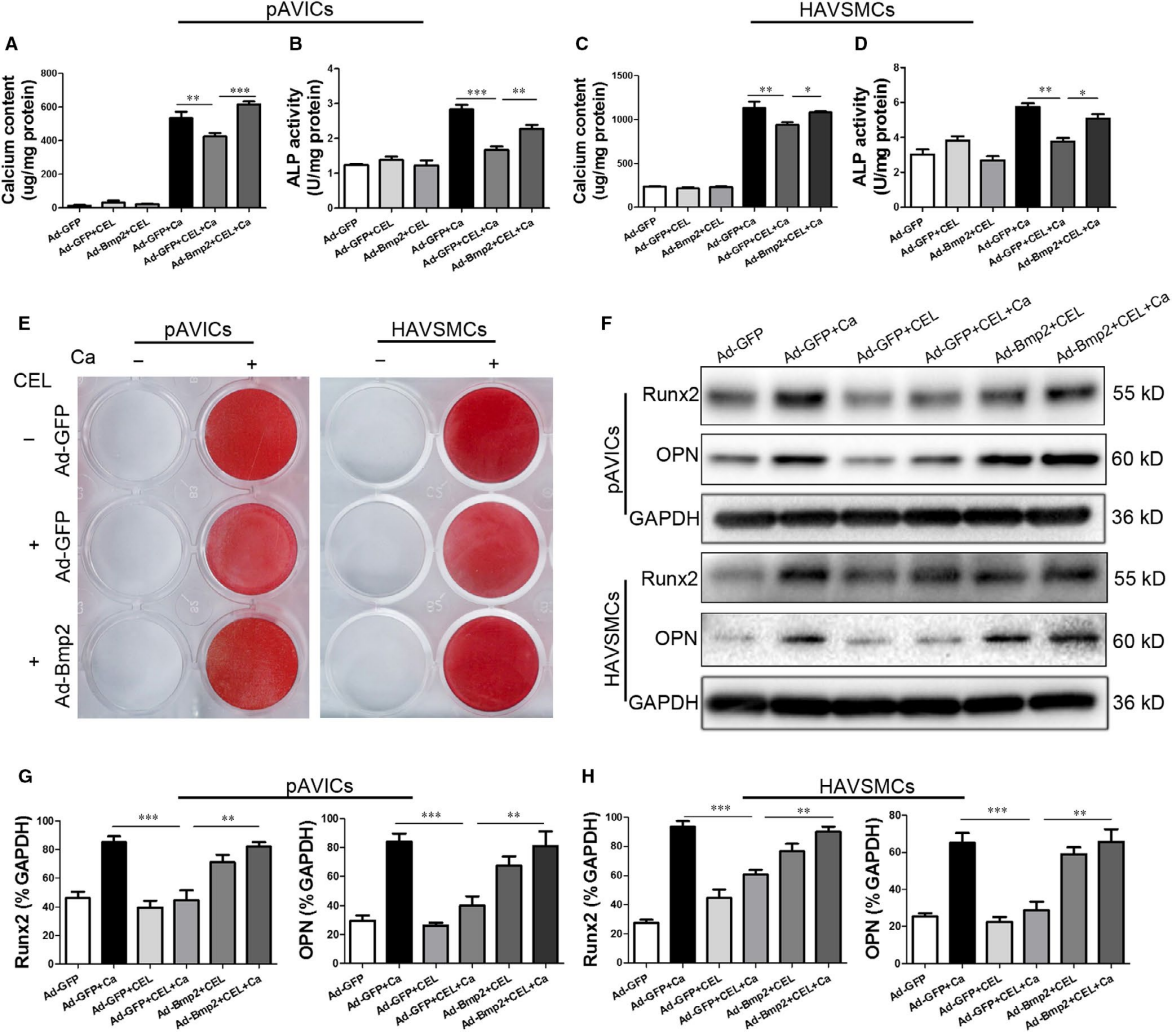
Overexpression of BMP2 reverses the anti‐calcification effect of CEL. A and C, Calcium content measured after overexpression of BMP2 using the QuantiChrom™ Calcium Assay Kit. B and D, Alkaline phosphatase (ALP) activity assessed after overexpression of BMP2 using the LabAssay™ ALP Kit. E, Alizarin red staining of cellular calcium deposition after overexpression of BMP2. F, Western blotting of Runx‐2 and OPN expression in pAVICs and HAVSMCs transfected with BMP2. G and H, Quantitative analysis of Runx2 and OPN expression. GAPDH served as the standard. All data are represented as mean ± SEM of at least three independent experiments. *P < .05, **P < .01 and ***P < .001 indicate significant differences between the indicated columns

**FIGURE 6:**
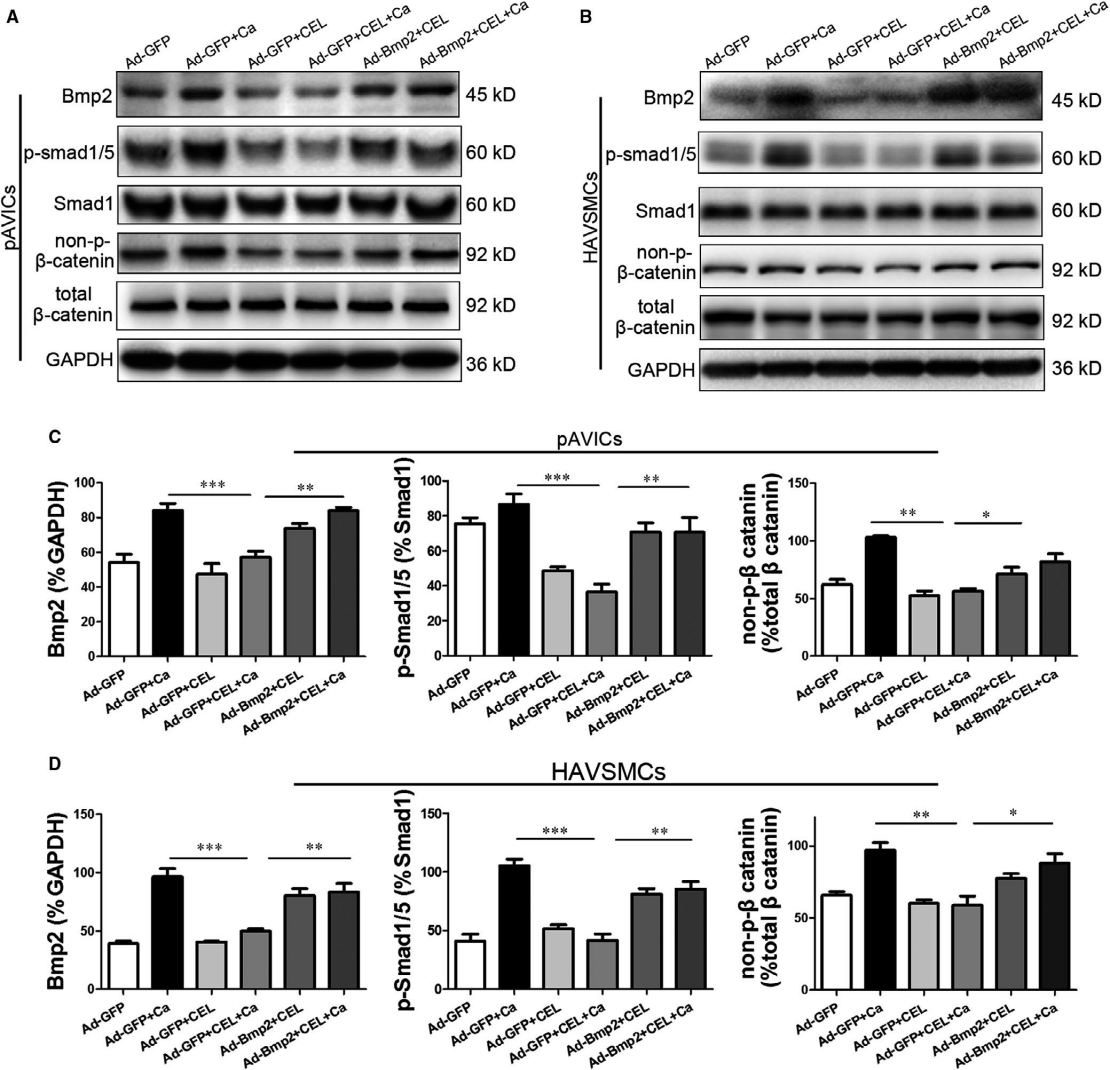
Overexpression of BMP2 reverses the expression of related proteins. A and B, Western blotting of BMP2, p‐Smad1/5, Smad1, non‐p‐β‐catenin and total β‐catenin expression in pAVICs and HAVSMCs transfected with BMP2. C and D, Quantitative analysis of protein expression. GAPDH served as the standard. All data are represented as mean ± SEM of at least three independent experiments. *P < .05, **P < .01 and ***P < .001 indicate significant differences between the indicated columns

To further explore whether the anti‐calcification effect of CEL depends on BMP2 signalling pathway, we overexpressed BMP2 in pAVICs using an adenovirus BMP2 vector. qRT‐PCR analysis showed that 3ⅹ10^7^ PFU/ml Ad‐BMP2 had the highest transfection efficacy (Figure [Link], [Link]A). Overexpression of BMP2 reversed the decrease of calcium content and alkaline phosphatase (ALP) activity in pAVICs (Figure 5A,B) and HAVSMCs (Figure 5C,D) reduced by CEL. Alizarin red staining, Western blotting and qRT‐PCR showed that the alleviation of cellular calcium deposition and Runx2 and OPN levels caused by CEL was blunted by Ad‐BMP2 treatment (Figure 5E‐H, Figure [Link], [Link]B,C). Then, Western blot results demonstrated that BMP2 overexpression reversed the CEL‐mediated decrease of BMP2, non‐p‐β‐catenin and p‐Smad1/5 both in pAVICs (Figure 6A,C) and in HAVSMCs (Figure 6B,D). These findings support that CEL attenuated calcification in pAVICs and HAVSMCs by inhibiting BMP2‐Smad1/5 and associated Wnt/β‐catenin pathways.

### 3.5 CEL down‐regulated the expression of BMPRII and up‐regulated the expression of Smad6

**FIGURE 7:**
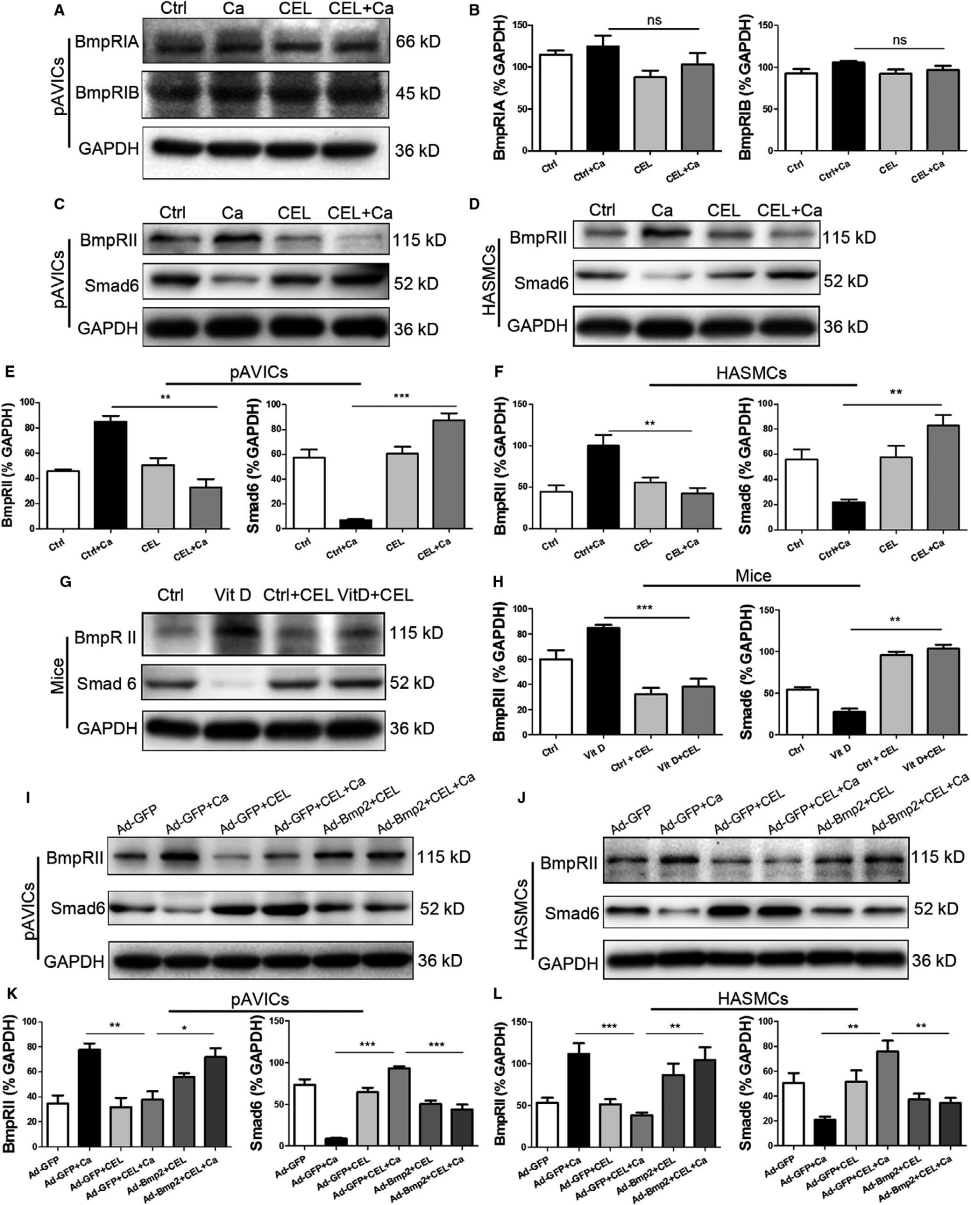
CEL down‐regulated BMPRII expression and activated Smad6 expression. A, Western blotting of BMPRIA and BMPRIB expression in pAVICs. B, Quantitative analysis of protein expression. GAPDH served as the standard. C and D, Western blotting of BMPRII and Smad6 expression in pAVICs and HAVSMCs. E and F, Quantitative analysis of protein expression. GAPDH served as the standard. G and H, Western blotting of BMPRII and Smad6 expression in mice. GAPDH served as the standard. I and J, Western blotting of BMPRII and Smad6 expression in pAVICs and HAVSMCs after overexpression of BMP2. K and L, Quantitative analysis of protein expression. GAPDH served as the standard. All data are represented as mean ± SEM of at least three independent experiments. *P < .05, **P < .01 and ***P < .001 indicate significant differences between the indicated columns

BMP2‐Smad signalling is related to type I receptor (BMP Receptor IA and IB (BMPRIA, BMPRIB)) and type II receptor (BMP Receptor II, BMPRII).^29^ We further examined the expression of BMPRIA and BMPRIB in pAVICs and found CEL treatment has no effect on the expression of BMPRIs (Figure 7A,B). However, the increase of BMPRII induced by CM was inhibited by the CEL treatment in pAVICs (Figure 7C,E) and HAVSMCs (Figure 7D,F). Recently, some previous studies demonstrated that Smad6 inhibits BMP‐Smad1/5 signalling and Wnt/β‐catenin signalling.^11^, ^30^ Therefore, the effect of CEL on Smad6 was examined, and we found that CEL prevented the decrease of Smad6 induced by CM in pAVICs (Figure 7C,E) and HAVSMCs (Figure 7D,F). The expression change of BMPRII and Smad6 in aortic artery in mice was consistent with the results in cell experiments (Figure 7G,H). As expected, overexpression of BMP2 almost entirely reversed the inhibition of BMPRII up‐regulation caused by CEL and partially reversed the preservation of Smad6 by CEL in pAVICs (Figure 7I,K) and HAVSMCs (Figure 7J,L). These results suggest that the change of BMPRII and Smad6 induced by BMP2 and CM can be regulated by CEL.

**FIGURE 8:**
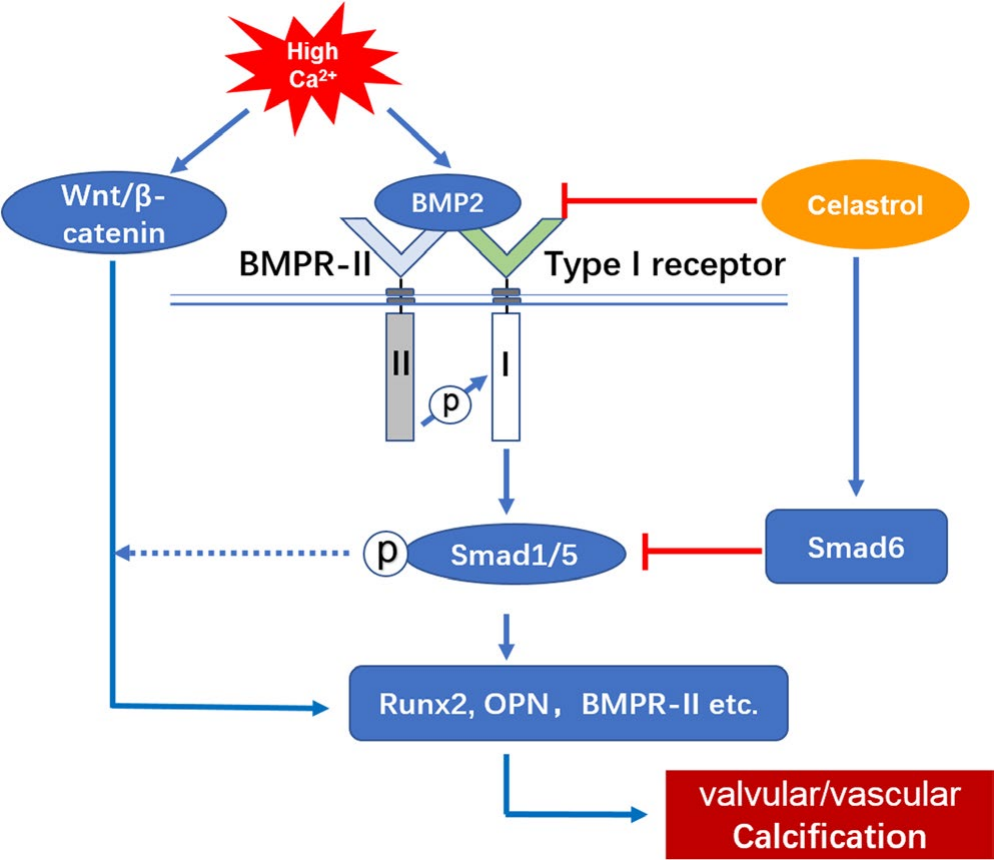
The possible signalling axis involved in the anti‐calcification effect of CEL. Schematic illustration of the potential molecules related to BMP2‐Smad1/5 and Wnt/β‐catenin signalling pathways

We for the first time illustrate that CEL prevents valvular/vascular calcification induced by high calcium via inhibiting BMP2‐Smad1/5 and Wnt/β‐catenin signalling pathway (Figure 8). This inhibition of calcification is further enhanced by blocking the up‐regulation of BMPRII and down‐regulation of Smad6 induced by high calcium (Figure 8).

## 4 DISCUSSION

Our major findings include the following: 1. CEL is a natural herbal compound with anti‐calcification effect at a low dose with nephrotoxicity. 2. CEL attenuates high calcium–induced aortic artery and aortic valve calcification in mice with renal dysfunction. 3. CEL attenuates the osteogenic differentiation of AVICs and VSMCs induced by high calcium by inhibiting BMP2‐mediated p‐Smad1/5 signalling. 4. CEL down‐regulates the expression of BMPRII level and maintains the expression of Smad6, which counteracts with the pro‐osteogenic effect produced by BMP2.

Extracts from some Chinese traditional medicine herbs including Puerariae radix, Cuscuta chinensis, Icariin and Drynaria fortunei are reported to promote osteogenic differentiation and may be helpful for the treatment of bone fracture. However, a few studies found that some of these natural compounds also have a protecting effect against vascular calcification. Recent studies have demonstrated that the extract of actinomycetes fermentation broth may provide a novel treatment for vascular calcification by inhibiting the expression of osteogenic genes, the VSMC oxidative stress and apoptosis rate.^31^, ^32^ In this study, we chose the CEL as a candidate natural compound considering clinically its long‐term use in China. Root extracts from TGV are routinely used in the treatment of rheumatoid arthritis and are found to be highly effective.^33^ Triptolide and CEL are the most extensively studied compounds extracted from TGV. In the marketed TGV products in China, the content of triptolide is used as a unique standard for the evaluation of drug quality. Previously, no significant protective effect of TGV on vascular calcification has been reported in the clinical practice. Recently, a study showed that triptolide as a NF‐κB inhibitor attenuates high‐phosphorus diet–induced artery calcification in a mouse CKD model.^34^

However, severe side‐effects of TGV or triptolide, especially nephrotoxicity, greatly limit their use, and the inhibitory effect of TGV on vascular calcification might counteract with the drug toxicity. Meanwhile, TGV is mainly used in those patients without significant renal dysfunction, but vascular calcification hardly occurred in this population. We think the beneficial effect of TGV should be based on one compound with a low nephrotoxicity. Although the molecular structure of CEL is different from triptolide, both of them have a strong anti‐inflammation effect. Therefore, we try to explore the anti‐calcification effect of CEL at a lower dose with minimized nephrotoxicity. Our data show that CEL at low dose only has a minor effect on cell viability, but produced a significant anti‐calcification effect on both VSMCs and AVICs. Also, in our mice with existed renal dysfunction, CEL has almost no influence on blood creatinine, although there is a moderate increase of BUN. Surprisingly, after the induction of vascular calcification using Vit D, both BUN and creatinine are decreased in mice treated with CEL, indicating that CEL might have a potential renal‐protecting effect. Overall, our data indicated that CEL might be a promising agent for the treatment of vascular calcification, and compared to triptolide, it has less negative effect on renal function.

Previous studies clearly illustrate that CEL can modulate multiple cell signalling pathways involved in inflammation and cancer (reviewed by Kannaiyan^15^). The major cell signalling pathways modulated by CEL include the JAK/STAT pathway, MAPK pathway, NF‐kB pathway, Notch‐1 pathway, RANKL/OPG pathway, PI3K/Akt/mTOR pathway and antioxidant defence mechanisms.^15^ However, the NF‐κB signalling pathway is thought to be the target of CEL.^35^, ^36^ NF‐κB pathway is highly activated in atherosclerosis,^37^, ^38^ but whether it also involves in the progression of vascular calcification is not clear. In high‐phosphorus diet–induced vascular calcification, inhibition of NF‐κB suppressed osteogenic differentiation of VSMC in a DBA/2 mouse CKD model.^34^ However, knockout of IKKβ, an important kinase for NF‐κB activation, was found to promote CaCl_2_‐induced vascular calcification in C57/BL6 mice.^39^ These contradictory results of NF‐κB signalling during calcification formation indicate the complicated mechanism involved in calcification, and the role of NF‐κB may be context‐dependent. In this study, we found that the phosphorylation of p65 and IκB was not induced by high calcium, and CEL also had no significant influence on their expression. Therefore, our data indicate that the activation of NF‐κB pathway is not important in the calcification of isolated AVICs. We build up a Vit D‐induced vascular calcification model using C57/BL6 mice with established renal dysfunction, which is different from the models previously reported,^34^ and our different results about NF‐κB suggest the complicated roles of NF‐κB in vascular calcification. In brief, our results indicated that targeting NF‐κB signalling pathway might not be an effective treatment for vascular calcification.

The negative results in NF‐κB pathways made us to explore the potential effect of CEL on other signalling pathways, such as Wnt/β‐catenin and BMP2/Smad1/5 pathways, which are important for bone formation and vascular calcification. Inactivation of Wnt signalling leads to constant phosphorylation of β‐catenin and the following degradation of phosphorylated β‐catenin.^40^ In contrary, activation of Wnt pathway results in stabilization of non‐phosphorylated β‐catenin and subsequent nuclear translocation.^40^ We found that CEL significantly reduced the expression of BMP2, p‐Smad1/5 and non‐phosphorylated β‐catenin both in vitro and in vivo. And in our study, for the first time CEL was found to inhibit Wnt and BMP2/Smad1/5 pathways. BMPs synergizes with Wnt/β‐catenin to regulate osteogenic differentiation, which is critical in promoting bone anabolism and remodelling.^28^ In our rescue experiment using recombinant BMP2 to antagonize the effects of CEL, we found that the overexpression of BMP2 recovered the non‐phosphorylated β‐catenin, which confirmed the Wnt activation mediated by BMP2 during vascular calcification. Considering that the total expression level of β‐catenin did not change with or without high calcium treatment, and CEL significantly inhibited the high calcium–induced BMP2 expression, our data further support the essential role of BMP2 signalling in initiating the osteogenic differentiation, and BMP2 plays a vital role in high calcium–induced vascular calcification.

There are two different BMP receptors, BMPRI and BMPRII, and BMP2 shows a high affinity for BMPRI and a low affinity for BMPRII. Binding of BMP2 to BMP receptors is the initial step of BMP signalling, and the expression level of BMP receptors can be regulated under some circumstances.^41^ Therefore, we examined the expression level of BMP receptors. Interestingly, we found that CEL had no significant effect on the expression of the high‐affinity BMPRI, but inhibited the increased expression of low‐affinity BMPRII induced by high‐calcium medium, which suggests that CEL inhibits BMP2 signalling in a stress‐based situation and may not affect the physiological function of VSMCs and AVICs. BMP2 can either directly bind to a preformed BMPRIA/BMPRII complex, or bind to BMPRIA at first, which results in the recruitment of BMPRII and formation of BMPRIA/BMPRII. The direct binding triggers the clathrin‐dependent internalization and then the activation of classical Smad signalling, whereas the “indirect” binding activates non‐Smad signalling, for example mitogen‐activated protein kinase (MAPK) signalling through caveolae‐mediated internalization. Our data showed that CEL inhibited the expression of both p‐p38 and p‐Smad1/5, which suggest a completely interruption of BMP2 signalling by CEL. Smad6, an inhibitory Smad, plays an important role in the progression of in vascular calcification.^42^ In our in vitro and in vivo calcification models, the expression of Smad6 was significantly inhibited after high‐calcium treatment. Also, CEL prevented this inhibition of Smad6, and overexpression of BMP2 partially reversed the preservation of Smad6 by CEL. In the atherosclerotic calcification model, TNF‐alpha was found to be related to the increased expression of BMP2 and decreased Smad6.^43^ TNF‐alpha directly activates NF‐κB signalling, and the preservation of Smad6 cannot be explained by the inhibition of NF‐κB pathway. CEL can also act as a proteasome inhibitor and induces apoptosis of cancer cells.^44^ However, a potent proteasome inhibitor MG‐132 suppressed the expression Smad6 in gastric cancer TMK1 cells,^45^ which is opposite to our results. Therefore, the preservation of Smad6 by CEL in our calcification models cannot explain the inhibition of proteasome. The mechanisms of CEL‐induced Smad6 preservation in vascular calcification need to be further investigated.

## CONFLICT OF INTEREST

The authors confirm that they have no conflicts of interest.

## AUTHOR CONTRIBUTION

**zhongping Su:** Conceptualization (equal); Data curation (equal); Formal analysis (equal); Methodology (equal); Writing‐original draft (equal); Writing‐review & editing (equal). **Pengyu Zong:** Conceptualization (equal); Methodology (equal); Writing‐original draft (equal). **ji Chen:** Data curation (equal); Methodology (equal); Validation (equal). **Shuo Yang:** Investigation (equal); Methodology (equal); Validation (equal). **Yihui Shen:** Data curation (supporting); Formal analysis (supporting); Methodology (supporting). **Yan Lu:** Data curation (supporting); Methodology (supporting); Supervision (lead). **Chuanxi Yang:** Formal analysis (equal); Funding acquisition (equal); Validation (equal). **Xiangqing Kong:** Funding acquisition (lead); Project administration (lead); Resources (equal); Supervision (equal). **Yan‐Hui Sheng:** Methodology (lead); Project administration (supporting); Resources (equal); Supervision (equal). **Wei Sun:** Data curation (supporting); Formal analysis (supporting); Funding acquisition (equal); Project administration (supporting); Resources (equal); Supervision (equal); Visualization (equal).

## Supporting information

Fig S1

Fig S2

Fig S3

Fig S4

Fig S5

Fig S6

Fig S7

Fig S8

Supplementary Material

## Data Availability

The data are available in the manuscript or the supplementary materials.

## References

[1] Schlieper G , Hess K , Floege J , Marx N . The vulnerable patient with chronic kidney disease. Nephrol Dial Transplant. 2016;31:382‐390. 25744273 10.1093/ndt/gfv041

[2] Raggi P , Boulay A , Chasan‐Taber S , et al. Cardiac calcification in adult hemodialysis patients. A link between end‐stage renal disease and cardiovascular disease? J Am Coll Cardiol. 2002;39:695‐701. 11849871 10.1016/s0735-1097(01)01781-8

[3] Chen NX , Moe SM . Vascular calcification: pathophysiology and risk factors. Curr Hypertens Rep. 2012;14:228‐237. 22476974 10.1007/s11906-012-0265-8 PMC3959826

[4] Qunibi WY , Abouzahr F , Mizani MR , Nolan CR , Arya R , Hunt KJ . Cardiovascular calcification in Hispanic Americans (HA) with chronic kidney disease (CKD) due to type 2 diabetes. Kidney Int. 2005;68:271‐277. 15954917 10.1111/j.1523-1755.2005.00402.x

[5] Bellasi A , Kooienga L , Block GA , Veledar E , Spiegel DM , Raggi P . How long is the warranty period for nil or low coronary artery calcium in patients new to hemodialysis? J Nephrol. 2009;22:255‐262. 19384844

[6] Johnson RC , Leopold JA , Loscalzo J . Vascular calcification: pathobiological mechanisms and clinical implications. Circ Res. 2006;99:1044‐1059. 17095733 10.1161/01.RES.0000249379.55535.21

[7] Dhore CR , Cleutjens JP , Lutgens E , et al. Differential expression of bone matrix regulatory proteins in human atherosclerotic plaques. Arterioscler Thromb Vasc Biol. 2001;21:1998‐2003. 11742876 10.1161/hq1201.100229

[8] Hinton RB Jr , Lincoln J , Deutsch GH , et al. Extracellular matrix remodeling and organization in developing and diseased aortic valves. Circ Res. 2006;98:1431‐1438. 16645142 10.1161/01.RES.0000224114.65109.4e

[9] Abedin M , Tintut Y , Demer LL . Vascular calcification: mechanisms and clinical ramifications. Arterioscler Thromb Vasc Biol. 2004;24:1161‐1170. 15155384 10.1161/01.ATV.0000133194.94939.42

[10] Yip CY , Chen JH , Zhao R , Simmons CA . Calcification by valve interstitial cells is regulated by the stiffness of the extracellular matrix. Arterioscler Thromb Vasc Biol. 2009;29:936‐942. 19304575 10.1161/ATVBAHA.108.182394

[11] Hruska KA , Mathew S , Saab G . Bone morphogenetic proteins in vascular calcification. Circ Res. 2005;97:105‐114. 16037577 10.1161/01.RES.00000175571.53833.6c

[12] Canalis E , Economides AN , Gazzerro E . Bone morphogenetic proteins, their antagonists, and the skeleton. Endocr Rev. 2003;24:218‐235. 12700180 10.1210/er.2002-0023

[13] Broege A , Pham L , Jensen ED , et al. Bone morphogenetic proteins signal via SMAD and mitogen‐activated protein (MAP) kinase pathways at distinct times during osteoclastogenesis. J Biol Chem. 2013;288:37230‐37240. 24235143 10.1074/jbc.M113.496950 PMC3873576

[14] Venkatesha SH , Moudgil KD . Celastrol and its role in controlling chronic diseases. Adv Exp Med Biol. 2016;928:267‐289. 27671821 10.1007/978-3-319-41334-1_12 PMC8056454

[15] Kannaiyan R , Shanmugam MK , Sethi G . Molecular targets of celastrol derived from Thunder of God Vine: potential role in the treatment of inflammatory disorders and cancer. Cancer Lett. 2011;303:9‐20. 21168266 10.1016/j.canlet.2010.10.025

[16] Sethi G , Ahn KS , Pandey MK , Aggarwal BB . Celastrol, a novel triterpene, potentiates TNF‐induced apoptosis and suppresses invasion of tumor cells by inhibiting NF‐kappaB‐regulated gene products and TAK1‐mediated NF‐kappaB activation. Blood. 2007;109:2727‐2735. 17110449 10.1182/blood-2006-10-050807

[17] Guo L , Luo S , Du Z , et al. Targeted delivery of celastrol to mesangial cells is effective against mesangioproliferative glomerulonephritis. Nat Commun. 2017;8:878. 29026082 10.1038/s41467-017-00834-8 PMC5638829

[18] Allison AC , Cacabelos R , Lombardi VR , Alvarez XA , Vigo C . Celastrol, a potent antioxidant and anti‐inflammatory drug, as a possible treatment for Alzheimer's disease. Prog Neuropsychopharmacol Biol Psychiatry. 2001;25:1341‐1357. 11513350 10.1016/s0278-5846(01)00192-0

[19] Li H , Zhang YY , Huang XY , Sun YN , Jia YF , Li D . Beneficial effect of tripterine on systemic lupus erythematosus induced by active chromatin in BALB/c mice. Eur J Pharmacol. 2005;512:231‐237. 15840409 10.1016/j.ejphar.2005.02.030

[20] Li H , Zhang YY , Tan HW , Jia YF , Li D . Therapeutic effect of tripterine on adjuvant arthritis in rats. J Ethnopharmacol. 2008;118:479‐484. 18577440 10.1016/j.jep.2008.05.028

[21] Zhou YX , Huang YL . Antiangiogenic effect of celastrol on the growth of human glioma: an in vitro and in vivo study. Chin Med J. 2009;122:1666‐1673. 19719969

[22] Yang H , Chen D , Cui QC , Yuan X , Dou QP . Celastrol, a triterpene extracted from the Chinese "Thunder of God Vine," is a potent proteasome inhibitor and suppresses human prostate cancer growth in nude mice. Can Res. 2006;66:4758‐4765. 10.1158/0008-5472.CAN-05-4529 16651429

[23] Liu J , Lee J , Salazar Hernandez MA , Mazitschek R , Ozcan U . Treatment of obesity with celastrol. Cell. 2015;161:999‐1011. 26000480 10.1016/j.cell.2015.05.011 PMC4768733

[24] Cheng M , Wu G , Song Y , et al. Celastrol‐induced suppression of the MiR‐21/ERK signalling pathway attenuates cardiac fibrosis and dysfunction. Cell Physiol Biochem. 2016;38:1928‐1938. 27160852 10.1159/000445554

[25] Tang M , Cao X , Zhang K , et al. Celastrol alleviates renal fibrosis by upregulating cannabinoid receptor 2 expression. Cell Death Dis. 2018;9:601. 29789558 10.1038/s41419-018-0666-y PMC5964092

[26] Taylor PM , Allen SP , Yacoub MH . Phenotypic and functional characterization of interstitial cells from human heart valves, pericardium and skin. J Heart Valve Dis. 2000;9:150‐158. 10678389

[27] Kim DY , Park JW , Jeoung D , Ro JY . Celastrol suppresses allergen‐induced airway inflammation in a mouse allergic asthma model. Eur J Pharmacol. 2009;612:98‐105. 19356734 10.1016/j.ejphar.2009.03.078

[28] Krishnan V , Bryant HU , Macdougald OA . Regulation of bone mass by Wnt signaling. J Clin Investig. 2006;116:1202‐1209. 16670761 10.1172/JCI28551 PMC1451219

[29] Sieber C , Kopf J , Hiepen C , Knaus P . Recent advances in BMP receptor signaling. Cytokine Growth Factor Rev. 2009;20:343‐355. 19897402 10.1016/j.cytogfr.2009.10.007

[30] Hegarty SV , O'Keeffe GW , Sullivan AM . BMP‐Smad 1/5/8 signalling in the development of the nervous system. Prog Neurogibol. 2013;109:28‐41. 10.1016/j.pneurobio.2013.07.002 23891815

[31] Salimi F , Hamedi J , Motevaseli E , Mohammadipanah F . Isolation and screening of rare Actinobacteria, a new insight for finding natural products with antivascular calcification activity. J Appl Microbiol. 2018;124:254‐266. 28990259 10.1111/jam.13605

[32] Salimi F , Jafari‐Nodooshan S , Zohourian N , Kolivand S , Hamedi J . Simultaneous anti‐diabetic and anti‐vascular calcification activity of Nocardia sp. UTMC 751. Lett Appl Microbiol. 2018;66:110‐117. 29223135 10.1111/lam.12833

[33] Canter PH , Lee HS , Ernst E . A systematic review of randomised clinical trials of Tripterygium wilfordii for rheumatoid arthritis. Phytomedicine. 2006;13:371‐377. 16487688 10.1016/j.phymed.2006.01.010

[34] Yoshida T , Yamashita M , Horimai C , Hayashi M . Smooth muscle‐selective nuclear factor‐kappaB inhibition reduces phosphate‐induced arterial medial calcification in mice with chronic kidney disease. J Am Heart Assoc. 2017;6:e007248. 29146611 10.1161/JAHA.117.007248 PMC5721793

[35] Vallabhapurapu S , Karin M . Regulation and function of NF‐kappaB transcription factors in the immune system. Annu Rev Immunol. 2009;27:693‐733. 19302050 10.1146/annurev.immunol.021908.132641

[36] Karin M . Nuclear factor‐kappaB in cancer development and progression. Nature. 2006;441:431‐436. 16724054 10.1038/nature04870

[37] Kutuk O , Basaga H . Inflammation meets oxidation: NF‐kappaB as a mediator of initial lesion development in atherosclerosis. Trends Mol Med. 2003;9:549‐557. 14659470 10.1016/j.molmed.2003.10.007

[38] Pamukcu B , Lip GY , Shantsila E . The nuclear factor–kappa B pathway in atherosclerosis: a potential therapeutic target for atherothrombotic vascular disease. Thromb Res. 2011;128:117‐123. 21636112 10.1016/j.thromres.2011.03.025

[39] Al‐Huseini I , Ashida N , Kimura T . Deletion of IkappaB‐kinase beta in smooth muscle cells induces vascular calcification through beta‐catenin‐runt‐related transcription factor 2 signaling. J Am Heart Assoc. 2018;7:e007405. 29301759 10.1161/JAHA.117.007405 PMC5778968

[40] Matthijs Blankesteijn W , Hermans KC . Wnt signaling in atherosclerosis. Eur J Pharmacol. 2015;763:122‐130. 25987418 10.1016/j.ejphar.2015.05.023

[41] Knaus P , Sebald W . Cooperativity of binding epitopes and receptor chains in the BMP/TGFbeta superfamily. Biol Chem. 2001;382:1189‐1195. 11592400 10.1515/BC.2001.149

[42] Galvin KM , Donovan MJ , Lynch CA , et al. A role for smad6 in development and homeostasis of the cardiovascular system. Nat Genet. 2000;24:171‐174. 10655064 10.1038/72835

[43] Li X , Lim J , Lu J , Pedego TM , Demer L , Tintut Y . Protective role of Smad6 in inflammation‐induced valvular cell calcification. J Cell Biochem. 2015;116:2354‐2364. 25864564 10.1002/jcb.25186 PMC5003537

[44] Yang H , Landis‐Piwowar KR , Chen D , Milacic V , Dou QP . Natural compounds with proteasome inhibitory activity for cancer prevention and treatment. Curr Protein Pept Sci. 2008;9:227‐239. 18537678 10.2174/138920308784533998 PMC3303152

[45] Wu WK , Sung JJ , Yu L , Cho CH . Proteasome inhibitor MG‐132 lowers gastric adenocarcinoma TMK1 cell proliferation via bone morphogenetic protein signaling. Biochem Biophys Res Comm. 2008;371:209‐214. 18435911 10.1016/j.bbrc.2008.04.059

