# Supplementary figures and images for: Celastrol attenuates arterial and valvular calcification via inhibiting BMP2/Smad1/5 signalling
Source: J Cell Mol Med. 2020 Sep 20;24(21):12476–90. doi: 10.1111/jcmm.15779 (PMC7686965; doi:10.1111/jcmm.15779)

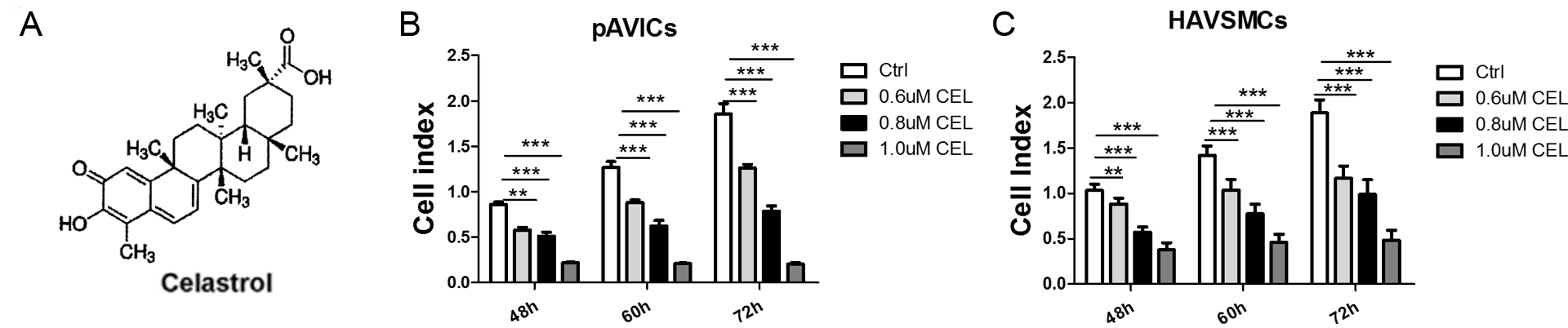

Supplement: Supplementary file 1 — Fig S1 [file JCMM-24-12476-s001.tif]

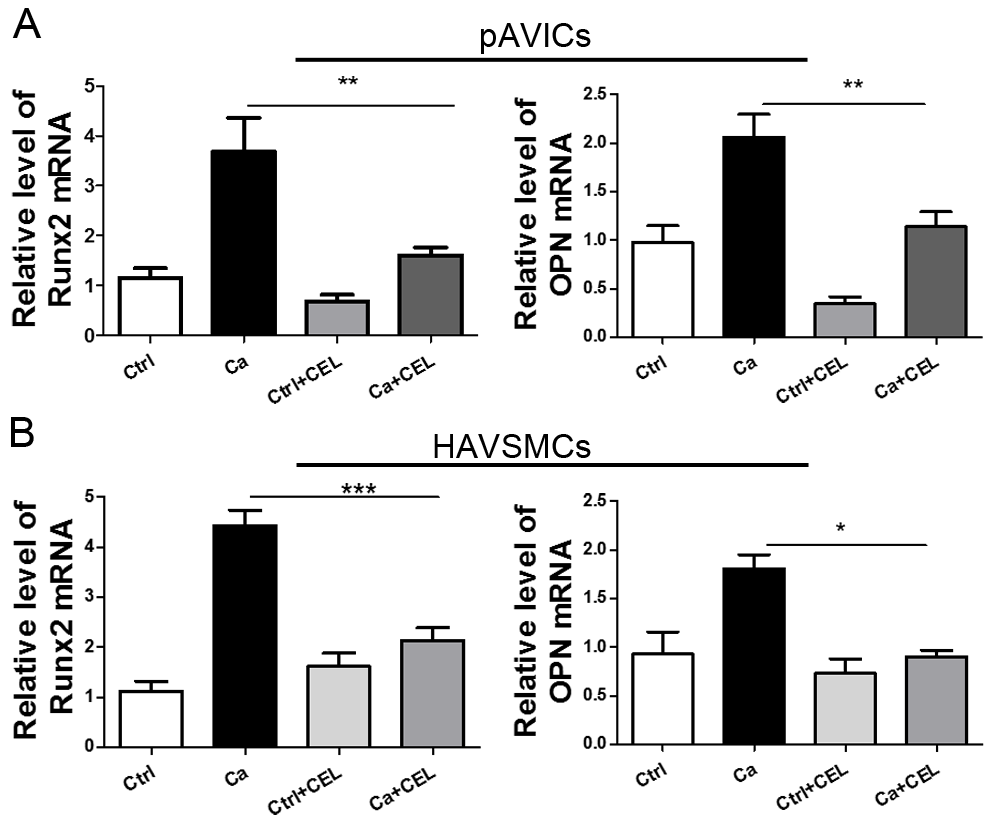

Supplement: Supplementary file 2 — Fig S2 [file JCMM-24-12476-s002.tif]

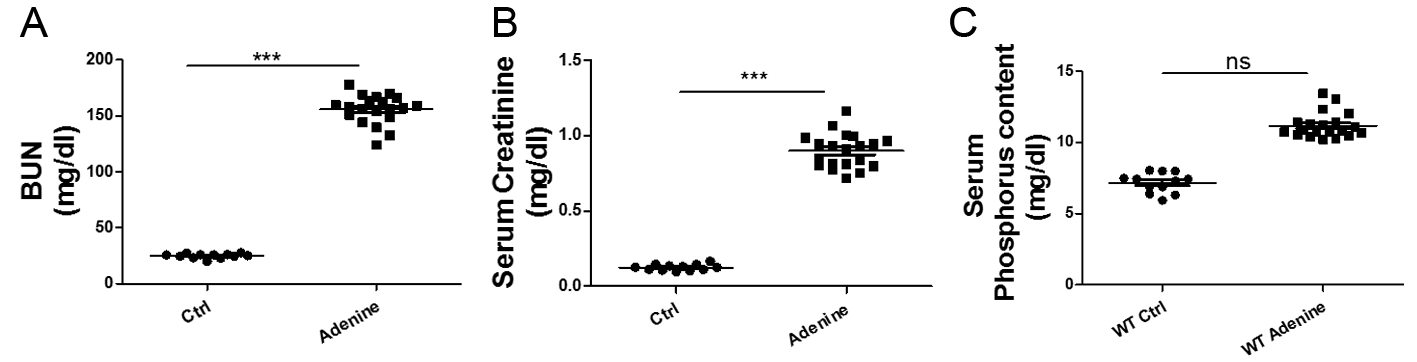

Supplement: Supplementary file 3 — Fig S3 [file JCMM-24-12476-s003.tif]

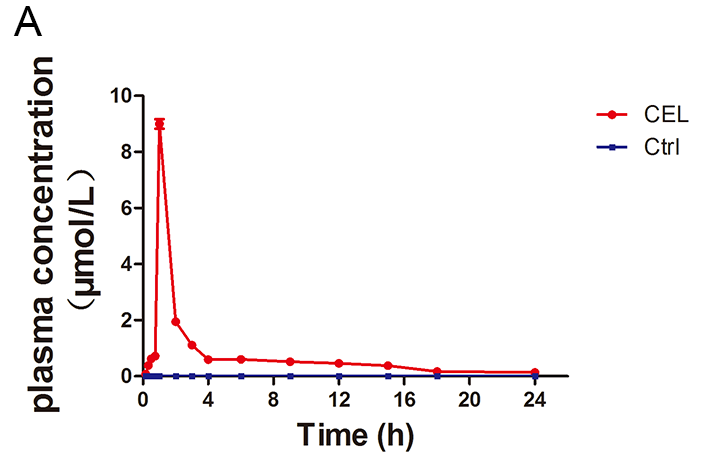

Supplement: Supplementary file 4 — Fig S4 [file JCMM-24-12476-s004.tif]

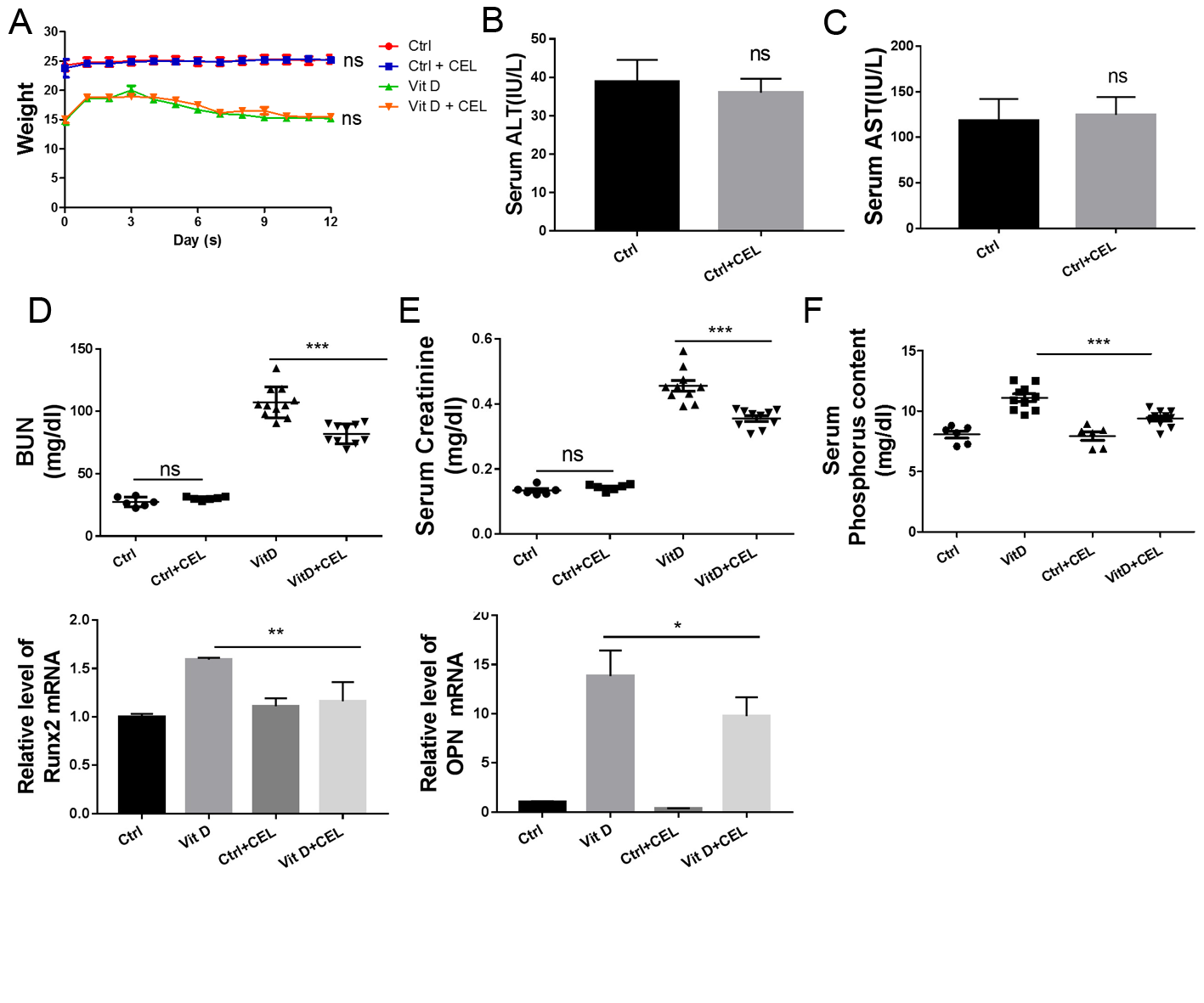

Supplement: Supplementary file 5 — Fig S5 [file JCMM-24-12476-s005.tif]

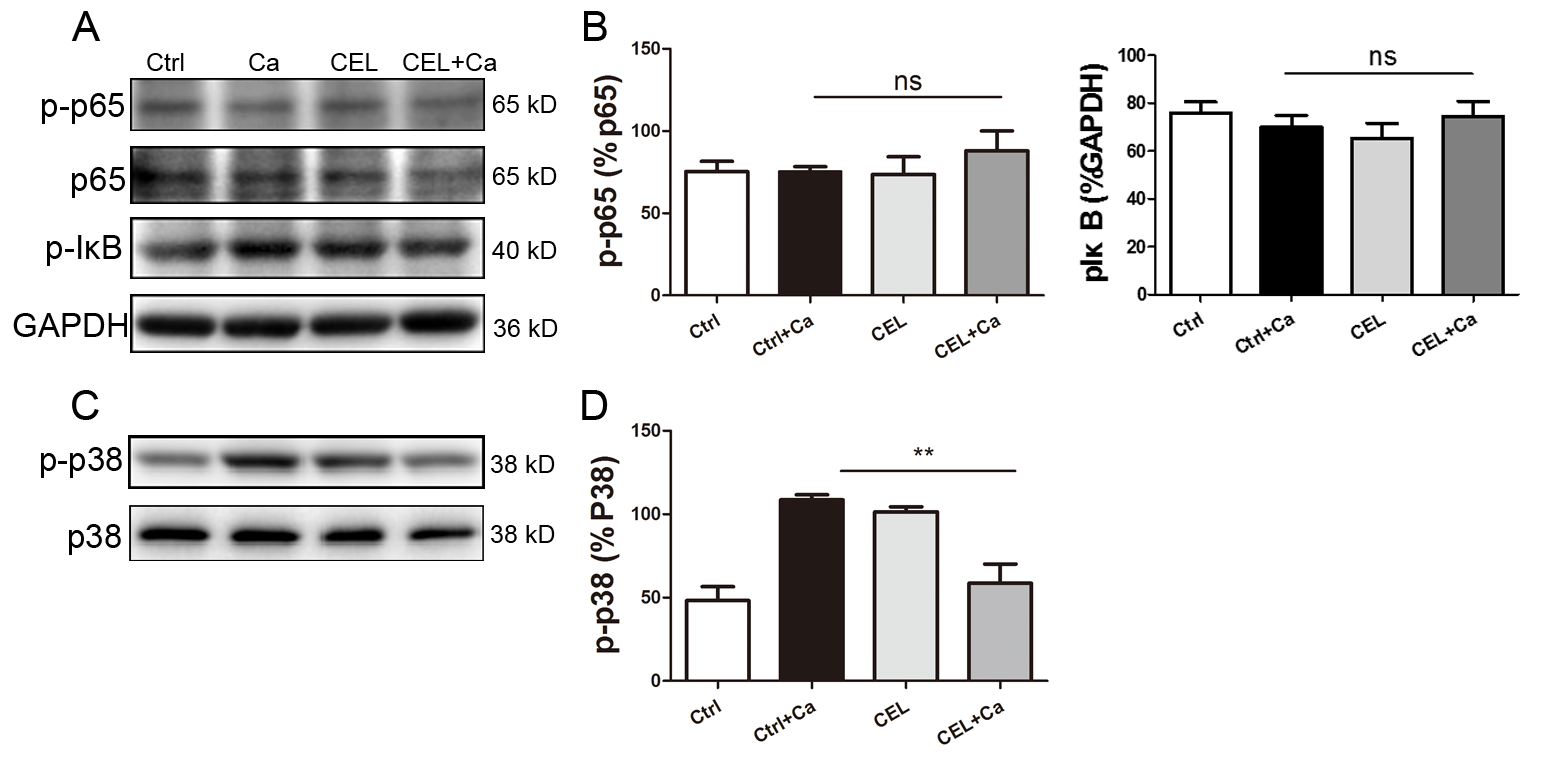

Supplement: Supplementary file 6 — Fig S6 [file JCMM-24-12476-s006.tif]

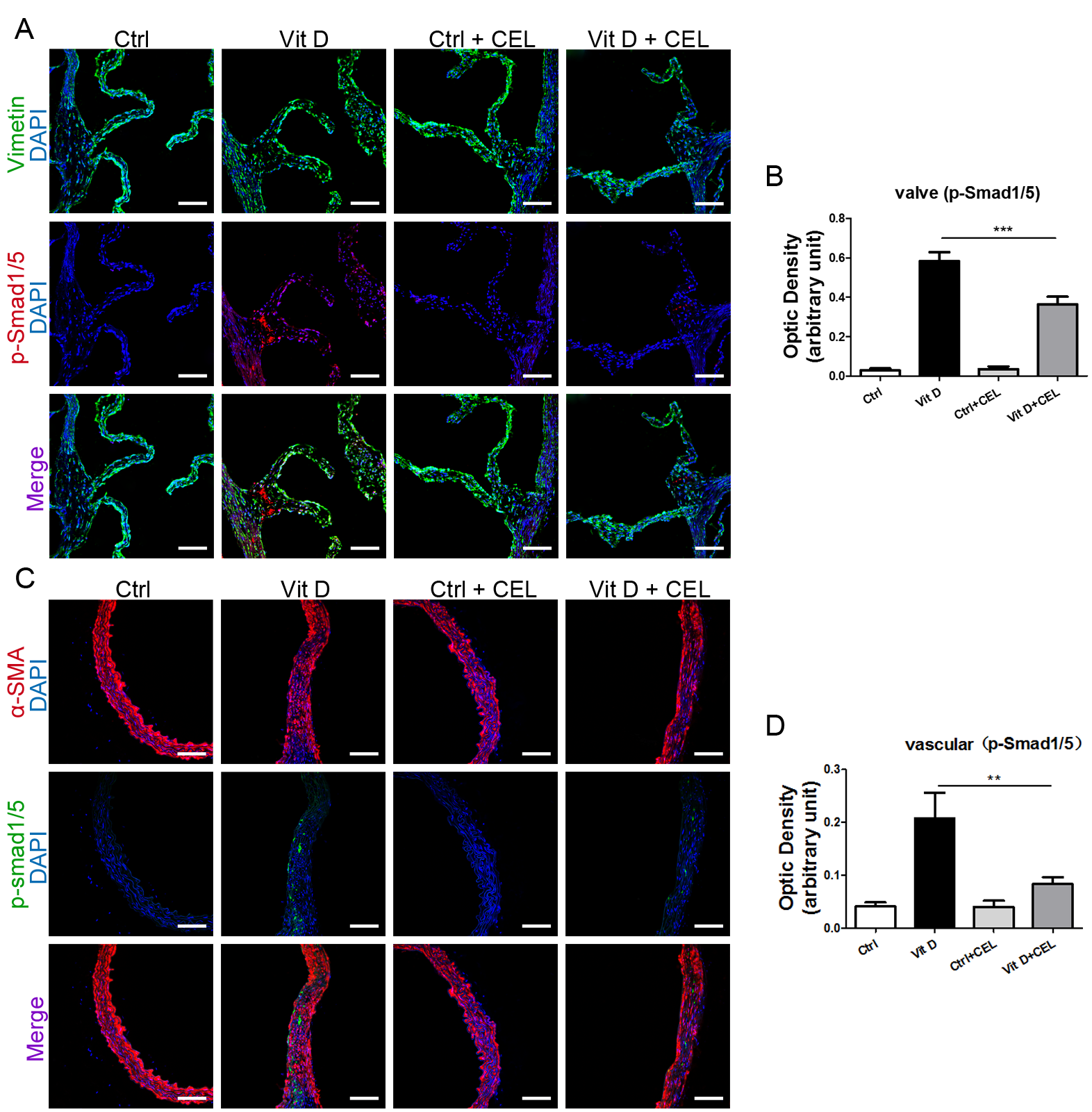

Supplement: Supplementary file 7 — Fig S7 [file JCMM-24-12476-s007.tif]

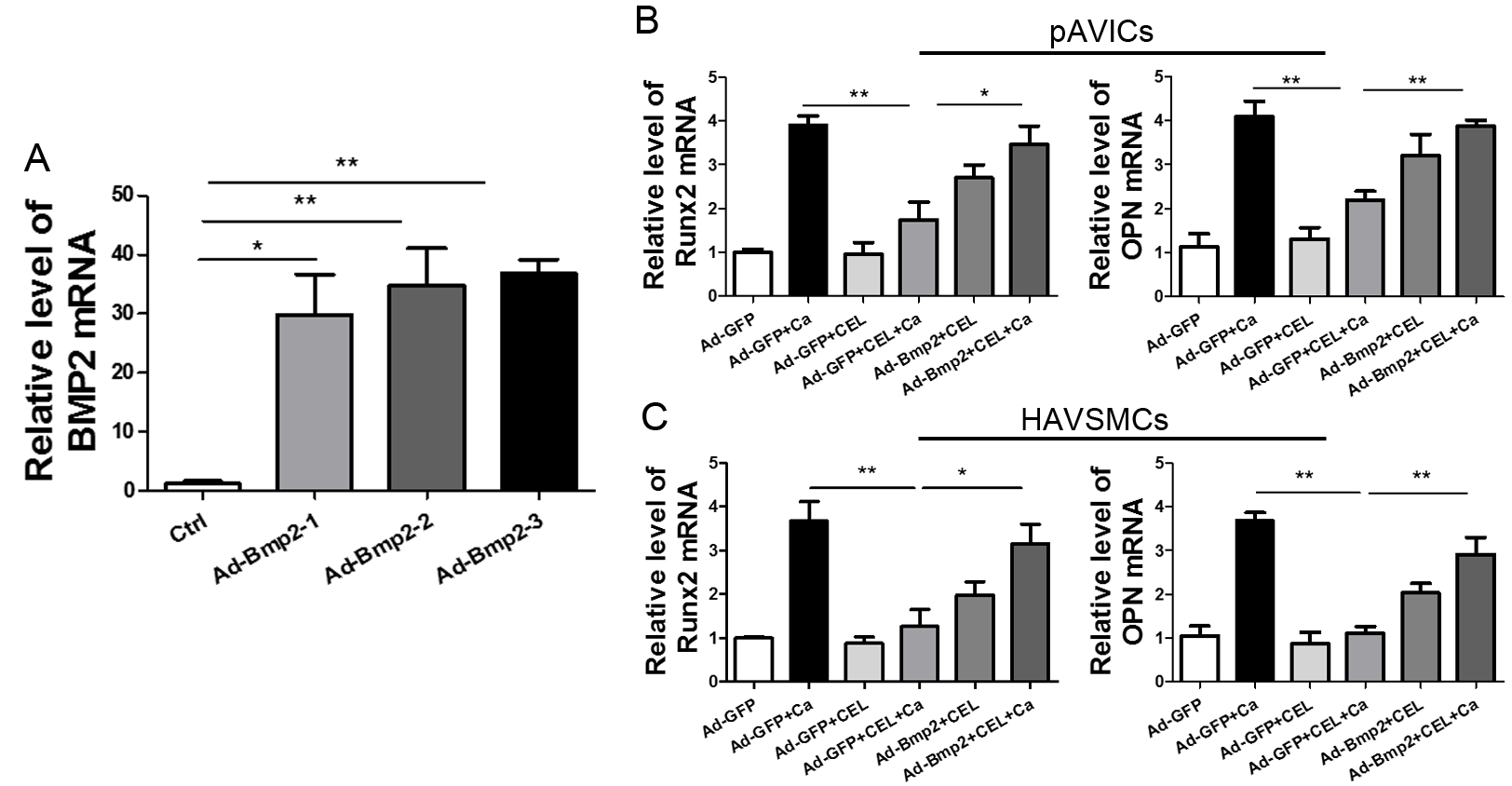

Supplement: Supplementary file 8 — Fig S8 [file JCMM-24-12476-s008.tif]
