# Supplementary material for: Celastrol attenuates arterial and valvular calcification via inhibiting BMP2/Smad1/5 signalling
Source: J Cell Mol Med. 2020 Sep 20;24(21):12476–90. doi: 10.1111/jcmm.15779 (PMC7686965; doi:10.1111/jcmm.15779)
Supplement: Supplementary file 9 — Supplementary Material [file JCMM-24-12476-s009.docx]

**Supplementary Fig. 1 Dose-dependent toxicity of CEL on pAVICs and HAVSMCs**

**A** Structure of celastrol. **B** Cell proliferation of pAVICs with CEL treatment at certain time points by RTCA. **C** Cell proliferation of HAVSMCs with CEL treatment at certain time points by RTCA. All data are represented as mean ± SEM of at least three independent experiments. **P<0.01 and ***P<0.001 indicate significant differences between the indicated columns.

**Supplementary Fig. 2 Expression of Runx2 and OPN mRNA in pAVICs and HAVSMCs cultured with CM**

**A** mRNA expression of Runx2 and OPN in pAVICs using qRT-PCR. **B** mRNA expression of Runx2 and OPN in HAVSMCs using qRT-PCR. The relative mRNA levels were normalized to GAPDH. All data are represented as mean ± SEM of at least three independent experiments. *P<0.05, **P<0.01 and ***P<0.001 indicate significant differences between the indicated columns.

**Supplementary Fig. 3 Monitoring of renal function after ending of adenine diet**

**A, B** Measurement of BUN and SCr of mice fed with adenine diet for 3 weeks. **C** Measurement of serum phosphorus concentration of mice fed with adenine diet for 3 weeks. (n=6-10 for each group) All data are represented as mean ± SEM of at least three independent experiments. ns indicates no significant difference between the indicated columns; ***P<0.001 indicate significant differences between the indicated columns.

**Supplementary Fig. 4 Plasma concentration of CEL in mice after subcutaneous injection.**

**A** Measurement of plasma concentration of CEL at different time points using liquid chromatography – mass spectrometry (LC-MS) technology.

**Supplementary Fig. 5 Monitoring of body weight, liver and renal function**

**A** Daily monitoring of body weight after the beginning of CEL administration. **B, C** Measurement of ALT and AST after injection of normal saline or CEL for 2 weeks. D**, E** Measurement of BUN and SCr of mice after injecting normal saline, vitamin D or CEL for 2 weeks. **F** Measurement of serum phosphorus concentration of mice after injecting normal saline, vitamin D or CEL for 2 weeks. (n=6-10 for each group) **G, H** mRNA expression of Runx2 and OPN in mice aortic artery using qRT-PCR. All data are represented as mean ± SEM of at least three independent experiments. ns indicates no significant difference between the indicated columns; *P<0.05, **P<0.01 and ***P<0.001 indicate significant differences between the indicated columns.

**Supplementary Fig. 6 p-p65, p-IκB and p-p38 expression in pAVICs treated with CM**

**A** Western Blotting of p-p65 and p-IκB expression. **B** Quantitative analysis of p-p65 and p-IκB expression. **C** Western Blotting of p-p38 expression in pAVICs. **D** Quantitative analysis of p-p38 expression. GAPDH served as the standard. All data are represented as mean ± SEM of at least three independent experiments. ns indicates no significant difference between the indicated columns. ns indicates no significant difference between the indicated columns; **P<0.01 indicate significant differences between the indicated columns.

**Supplementary Fig. 7 Expression of p-Smad1/5 in aortic valve and aortic artery of mice**

**A, B** Representative images of immunofluorescence staining of p-Smad1/5 in mice aortic valve (scale bar= 20µm, n=6~10 for each group). **C, D** Representative images of immunofluorescence staining of pSmad1/5 in mice aortic artery (scale bar= 20µm, n=6~10 for each group). All data are represented as mean ± SEM of at least three independent experiments. **P<0.01 and ***P<0.001 indicate significant differences between the indicated columns.

**Supplementary Fig. 8 Expression of Runx2 and OPN mRNA after overexpression of BMP2.**

**A** Three concentrations of adenovirus BMP2 vector, 1×107pfu/ml, 2×107pfu/ml, and 3×107pfu/ml, were used to induce BMP2 overexpression. mRNA expression of BMP2 using qRT-PCR after transfecting pAVICs. **B** mRNA expression of Runx2 and OPN in pAVICs using qRT-PCR. **C** mRNA expression of Runx2 and OPN in HAVSMCs using qRT-PCR. All data are represented as mean ± SEM of at least three independent experiments. *P<0.05 and **P<0.01 indicate significant differences between the indicated columns.
